# The application of machine learning algorithms for predicting length of stay before and during the COVID-19 pandemic: evidence from Wuhan-area hospitals

**DOI:** 10.3389/fdgth.2024.1506071

**Published:** 2024-12-13

**Authors:** Yang Liu, Renzhao Liang, Chengzhi Zhang

**Affiliations:** ^1^ School of Information Management, Wuhan University, Wuhan, China; ^2^ Shenzhen Research Institute, Wuhan University, Shenzhen, China; ^3^ School of Physics and Technology, Wuhan University, Wuhan, China; ^4^ Department of Information Management, Nanjing University of Science & Technology, Nanjing, China

**Keywords:** length of stay, COVID-19 pandemic, machine leaning, medical insurance, household registration

## Abstract

**Objective:**

The COVID-19 pandemic has placed unprecedented strain on healthcare systems, mainly due to the highly variable and challenging to predict patient length of stay (LOS). This study aims to identify the primary factors impacting LOS for patients before and during the COVID-19 pandemic.

**Methods:**

This study collected electronic medical record data from Zhongnan Hospital of Wuhan University. We employed six machine learning algorithms to predict the probability of LOS.

**Results:**

After implementing variable selection, we identified 35 variables affecting the LOS for COVID-19 patients to establish the model. The top three predictive factors were out-of-pocket amount, medical insurance, and admission deplanement. The experiments conducted showed that XGBoost (XGB) achieved the best performance. The MAE, RMSE, and MAPE errors before and during the COVID-19 pandemic are lower than 3% on average for household registration in Wuhan and non-household registration in Wuhan.

**Conclusions:**

Research finds machine learning is reasonable in predicting LOS before and during the COVID-19 pandemic. This study offers valuable guidance to hospital administrators for planning resource allocation strategies that can effectively meet the demand. Consequently, these insights contribute to improved quality of care and wiser utilization of scarce resources.

## 1 Introduction

The rapid global spread of the coronavirus disease (COVID-19) since 2019 has posed a significant threat to healthcare systems worldwide (1). One of the key challenges resulting from the surge in infections is the increased demand for hospital beds (2). However, hospital beds are limited; if the demand for beds exceeds hospital capacity, this will severely reduce the quality of care provided (3). For example, during the early stages of the outbreak, many people died in Wuhan due to infection because they were not admitted to hospital. Therefore, accurately predicting the demand for hospital beds is crucial to proactively expand capacity and indicate the effectiveness of public health interventions (4). Another important aspect to consider is the length of stay (LOS), which refers to the cumulative duration of a patient's hospitalization between consecutive admissions and discharges within a specific timeframe (5). Most hospitals face the challenge of providing timely patient care while maintaining optimal resource utilization, especially during the COVID-19 pandemic (6). We aim to forecast the LOS before and during the COVID-19 pandemic, considering the evolving circumstances caused by COVID-19. By predicting LOS, we can better understand and anticipate the resource requirements for efficient and effective healthcare services amidst the pandemic.

Previous research on factors influencing LOS has predominantly relied on traditional statistical models, simulation models, and other similar approaches (7). With the advancements in artificial intelligence techniques, machine learning has gained significant popularity in LOS prediction (8). However, LOS is a multifaceted metric impacted by various factors, such as individual demographics, diverse treatment strategies, and discharge plans, which can extend LOS beyond the target range (9). Hence, it is crucial to develop personalized and accurate LOS prediction models considering these influential factors. These models are pivotal in enhancing hospital resource allocation and informing healthcare decisions. In line with this objective, this paper focuses on analyzing the impact of medical insurance utilization on LOS for the residents of Wuhan during the COVID-19 pandemic, starting with examining medical insurance-related factors. By delving into the factors associated with medical insurance, we can gain valuable insights into how it affects LOS for individuals affected by the pandemic.

In 2005, the World Health Organization (WHO) encouraged its members to achieve the goal of universal health coverage, where all people have access to quality primary health care services, including promotion, prevention, treatment, rehabilitation, and palliative care, without having to endure financial hardship (10). In China, using the health care component of medical insurance supplements inadequate long-term care services (11). Due to the low quality of primary health care in China, patients have not used expensive tertiary hospitals (12). For this reason, many countries, including China, have taken adequate measures to expand their health coverage. Since good health care resources are concentrated in provincial capitals and most people from non-capital cities will visit provincial capitals, we will analyze the use of medical insurance by patient's household registration is in Wuhan compared to those in non-household registration is in Wuhan.

Several studies emphasize that insurance type and coverage significantly influence hospital LOS (13). Patients with private insurance often experience shorter stays due to streamlined discharge planning and access to post-acute care facilities. In contrast, those with public insurance or uninsured patients may face longer stays due to socioeconomic factors or limited post-discharge options (14). For example, Clarke et al. (15) observed that uninsured patients frequently experience delays in care transitions, leading to extended LOS. Hospital reimbursement models tied to insurance play a pivotal role in LOS. Prospective payment systems, such as diagnosis-related groups, incentivize hospitals to minimize LOS without compromising care quality. On the other hand, fee-for-service models may inadvertently encourage longer stays, as hospitals are compensated per service provided (16). These economic drivers underscore the necessity of predictive models that account for insurance-related factors to optimize LOS predictions.

Disparities in insurance coverage can lead to heterogeneity in LOS prediction outcomes. For instance, patients with inadequate insurance may exhibit irregular hospital utilization patterns, challenging algorithms' predictive accuracy. Toth et al. (17) highlight that integrating insurance status with social determinants of health in prediction models can improve their reliability, particularly for underserved populations. The COVID-19 pandemic has further illuminated the connection between LOS and insurance. Galvani et al. (18) revealed that uninsured or underinsured patients were less likely to seek early medical care during the pandemic, leading to increased severity at admission and, consequently, longer LOS. These findings suggest the need for dynamic models to adapt to such crises and reflect changes in healthcare utilization patterns (19). Understanding the interplay between insurance and LOS has critical policy implications. Policymakers can use insights from predictive models to design interventions that address inequities in healthcare access and optimize LOS management. For instance, targeted strategies to enhance post-acute care access for publicly insured or uninsured populations can reduce unnecessary hospital days and improve overall system efficiency.

To date, extensive research on COVID-19 has primarily centered around epidemiological investigations, diagnostics, treatments, and prevention and control strategies (20). However, there needs to be published studies explicitly focusing on the development of machine learning models to estimate LOS for COVID-19 patients in Wuhan throughout the three-year pandemic, which is the core focus of this study. Motivated by this research gap, the primary objective of this study was to estimate the duration of LOS for COVID-19 inpatients by utilizing fundamental hospitalization data. This involved analyzing a range of variables to create a comprehensive model that could predict LOS with greater accuracy. By doing so, the study aimed to provide valuable insights into the factors influencing the duration of hospitalization, which could help healthcare providers optimize resource allocation, improve patient management strategies, and enhance overall hospital efficiency during the ongoing pandemic.

The aim was to systematically compare different event onset time models for individualized LOS prediction, thus contributing to the efficient allocation of healthcare resources during the COVID-19 pandemic. To achieve this, we employed six distinct machine learning models, and their performance was thoroughly evaluated and compared. In summary, the research presented in this paper strives to accomplish the following: Firstly, this paper compared predictions made during non-pandemic periods with those made during pandemic periods, highlighting a shift in people's prioritization of their health status. Secondly, the primary objective of this study is to develop machine learning models that can effectively predict and estimate the duration of hospitalization for COVID-19 patients in Wuhan, which aid health authorities in efficiently managing their resources and providing crucial guidance for accepting new patients. Finally, this research aims to contribute to a deeper understanding of the interplay between medical insurance utilization and LOS, ultimately paving the way for improved resource management and more informed healthcare strategies.

## 2 Methods

### 2.1 Data collection and preprocessing

Our study was conducted after collecting electronic medical record data from Zhongnan Hospital of Wuhan University, which is the largest regional teaching hospital in Wuhan. Since the beginning of the pandemic, this hospital has been the leading center for receiving and treating COVID-19 patients from Wuhan. The data were collected from electronic medical queries to identify patient records. With China's full reopening in December 2022, the data were collected from January 2017 to December 2022, mainly reflecting before and during the COVID-19 pandemic. Data have been collected using a structured form. It contains clinical and demographic data for 158,854 patients from 2017 to 2019 and 217,970 patients who visited the hospital before COVID-19. We divided the data into Wuhan and non-Wuhan medical insurance because the reimbursement rate varies from place to place, and patients can only be reimbursed in their domicile.

Sample selection is an effective technique that is used to determine the most meaningful variables, reduce the dimensions of the dataset, and improve the efficiency of ML algorithms. In this study, we identified 35 variables that were valid on the electronic medical record as effective predictors of LOS in patients with COVID-19 before and during the COVID-19 pandemic. Each patient record contains 35 variables collected at admission and in the patient's medical history, and each patient's record is updated daily. These characteristics include a wide range of basic patient information, clinical and demographic data containing comorbidities, laboratory results, and symptoms, and detailed variables shown in Table 1. In contrast, the sea of data contains category-based variables, as specified in Table 2. For the altered data, we directly coded, for example, hospital departments, which have 91 categories, we directly coded from 1 to 91, and so on.

**Table 1:** Patient demographics and clinical characteristics.

Variables	Unit	Minimum	Median	Maximum	Mean	Variance
Age	Year	0.0	55.0	105.00	52.58	17.54
Less than 1 year of age	Year	0.0	0.0	11.5	0.01	0.24
Newborn birth weight	g	0.0	0.0	6,000	149.93	679.93
Newborn admission weight	g	0.0	0.0	9000.0	33.83	322.53
Account payment amount	RMB	−3835.5	0.0	94545.0	386.75	1365.15
Out-of-pocket amount	RMB	−6800.0	7793.52	1776393.38	13925.49	20798.21
Total surgical level	Level	0.0	4.0	72.0	6.1	5.2
Average surgical level	Level	0.0	2.33	6.0	2.37	0.92
Total healing level	Level	0.0	4.0	80.0	6.09	6.46
Average level of healing	Level	1.0	1.0	4.0	2.31	1.43
Total incision level	Level	0.0	1.0	45.0	2.07	2.84
Average level of incision	Level	0.0	1.0	3.0	0.8	0.81
Number of surgeries	Times	1	2.0	23	2.62	1.95
Number of diagnosed conditions	Times	2	6.0	32	6.49	3.03
Medical insurance	RMB	−6885.0	7470.96	1245234.9	15039.77	24334.6

**Table 2:** Categorical variables for patients.

Variables	Number of categories	Category description
Gender	2	Male or female
Admission department	91	Infectious Diseases Ward Infectious diseases ward, department of general medicine, department of traditional chinese medicine (integrated traditional chinese and western medicine) ward, hematology ward, etc.
Nationality	30	China, the United States, Spain, etc.
Ethnicity	33	Han, Mongolian, Hui, Tibetan, Uighur, Miao, etc
Career	15	Farmer, retired personnel, civil Servants, Other, etc.
Marriage	2	Married, unmarried
Place of origin-province	36	Hebei, Shanxi, Liaoning, Jilin, Heilongjiang, Jiangsu, Zhejiang, Anhui, Fujian, etc.
Blood type	5	Not checked, AB, A, Unknown, O, etc.
RH (Blood type)	5	Positive, not checked, negative
Rehospitalization program	5	To live, not to live, others, etc.
Current address	35	Wuhan, Huangshi, Yichang, Xiangyang, etc.
Household address	43	Wuhan, Huangshi, Yichang, Xiangyang, etc
Relationship between contact person and patient	95	Father and son, mother and son, dependents, none, etc.
Admission route	4	Direct admission, transfer, continued hospitalization, etc.
Allergy drug flagging	4	Allergenic, non-allergic, others, etc.
Quality of the first page of the case	4	Poor, average, excellent, etc.
Condition at admission	4	Poor, average, excellent, etc.
Type of surgical patient	4	Need surgery, no, other, etc.
Category of medical insurance	51	Municipal medical insurance, non-medical insurance, targeted poverty alleviation, others, etc.
Type of insurance	16	Ordinary hospitalization, extraordinary hospitalization, others, etc.

We also cleaned the data. Meanwhile, in Table 3, we also calculated the confidence intervals of each variable. Identify and remove duplicates in datasets to avoid bias and redundancy. Apply techniques like min-max normalization or z-score standardization to make data comparable. Correct misspellings, incorrect entries, or impossible values. Use smoothing techniques or data filters to eliminate unnecessary data variations. Ensure uniform formatting across datasets. We also handle missing values. Remove rows with missing values. Suitable for datasets with minimal missing data. Remove features with high percentages of missing data. Replace missing values with the feature's mean, median, or mode.

**Table 3:** Confidence intervals for each variable.

Variables	Bias-corrected confidence intervals (95%)		Percentile confidence interval (95%)	
	Lower limit	Upper limit	Lower limit	Upper limit
Age	0.0129,	0.1822	0.0076,	0.1869
Less than 1 year of age	0.0032	0.1890	−0.0035	0.1670
Newborn birth weight	−0.0022	0.1867,	−0.0080	0.1576
Newborn admission weight	0.0025	0.1847	−0.0104	0.1755
Account payment amount	0.0078	0.1730	0.0093	0.1595
Out-of-pocket amount	0.0153	0.1853	−0.0001	0.1687
Total surgical level	0.0113	0.1660	0.0043	0.1678
Average surgical level	−0.0005	0.1805	0.0044	0.1815
Total healing level	0.0160	0.1972	0.0004	0.1817
Average level of healing	0.0075	0.1992	−0.0251	0.1799
Total incision level	0.0057	0.1872	0.0159	0.1665
Average level of incision	0.0026	0.1887	0.0021	0.1814
Number of surgeries	0.0109	0.1862	0.0012	0.1694
Number of diagnosed conditions	0.0063	0.1876	0.0200,	0.1735
Medical insurance	0.0085	0.2082	−0.0008	0.1747

### 2.2 Machine learning methods

**Figure 1:**
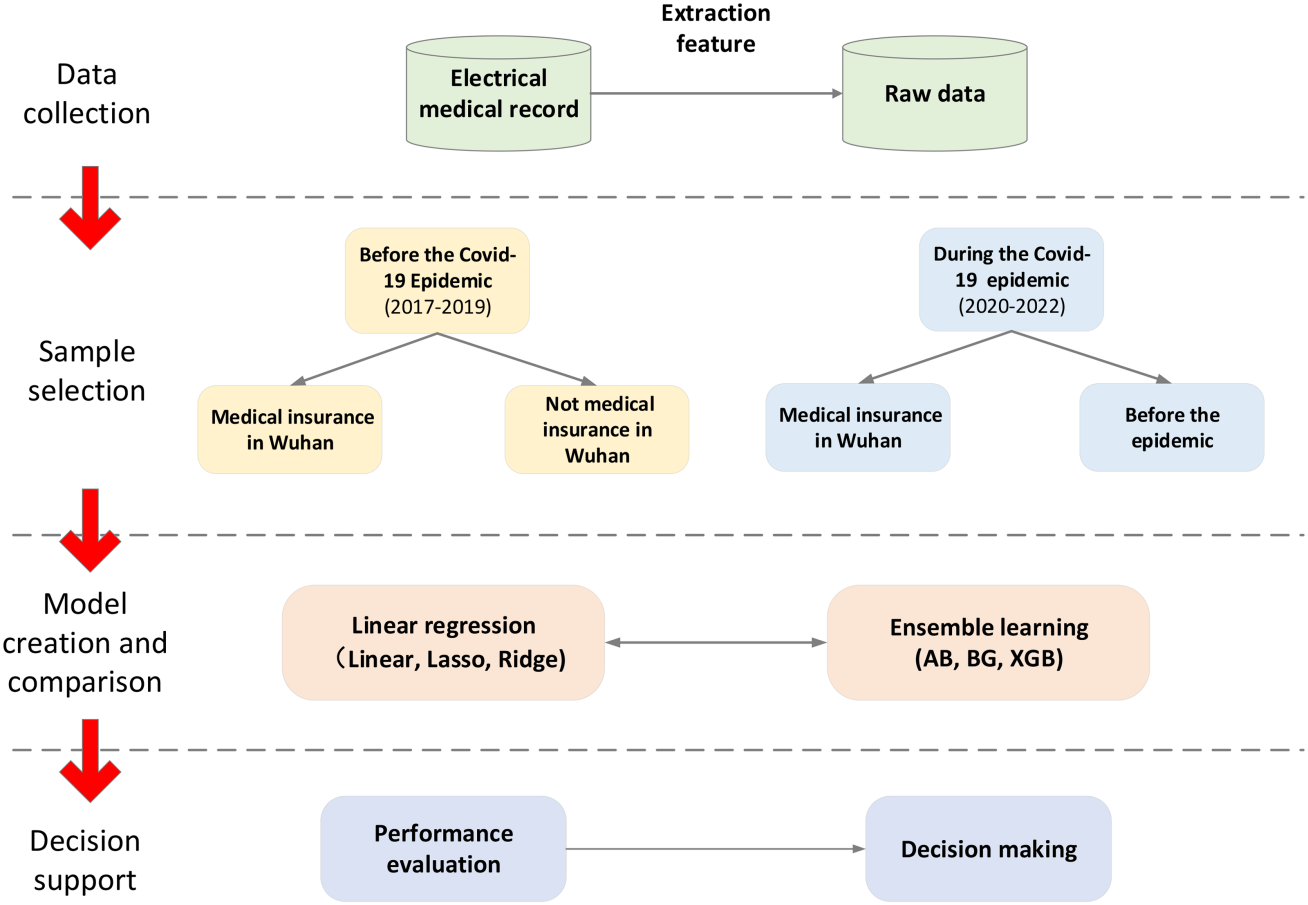
Architecture of proposed model.

In this study, three linear regression algorithms and three ensemble learning algorithms are used to predict LOS, and the different models' performance is compared. Figure 1 illustrates the steps of the proposed method. Thank you for your suggestion. We have added the section on data cleaning and missing values. The details are as follows:

#### 2.2.1 Linear regression algorithm

Three classical machine learning regression models were employed in this study before and during the COVID-19 pandemic prediction. The selected models include linear regression, linear lasso regression, and linear ridge regression. The advantage of these methods is that the modeling is fast, does not require very complex calculations, and still runs fast despite the large amount of data. Interpretation of each variable can be given based on the coefficients (21). Linear regression models are widely used in supervised machine learning and are particularly effective in identifying linear relationships to predict target attributes (22).

The parameters of the linear regression model (LR) (23) can be minimized using a flat method in the LASSO regression model (LA) (24), which is a type of linear regression known as “minimum absolute shrinkage and selection operator”. Similarly, the ridge regression (RR) model, a different kind of predictive regression model, utilizes coefficients to solve for standard linearity (25). It is worth noting that L1 regularization, used in Lasso regression, applies a penalty instead of the L2 regularization penalty employed in ridge regression. In this algorithm, we mainly use the scikit-learn package of python for analysis.

#### 2.2.2 Ensemble learning

Ensemble learning combines multiple weakly supervised models to obtain a more robust and comprehensive supervised model (26). The fundamental concept behind ensemble learning is that even if a weak classifier produces incorrect predictions, the collective wisdom of other weak classifiers can correct those errors (27). In this study, we will utilize the following three algorithms:

Bagging (BG) is a technique proposed by (28), that involves creating a new training set by randomly selecting a subset of training samples from the original set, allowing for replacement (put-back sampling). Each subset is then used to train a sub-model. By aggregating the predictions of these sub-models, we can achieve a more accurate and robust final forecast. AdaBoost (AB) is one Boosting (29), which is an important integrated learning technique that can enhance a weak learner with only slightly higher prediction accuracy than random guesses into a strong learner with high prediction accuracy. A new weak classifier is added in each iteration until a predefined small enough error rate is reached or a pre-specified maximum number of iterations is reached to determine the final strong classifier.

XGBoost (XGB) is a boosting algorithm based on Friedman's efficient and scalable implementation of gradient model boosting (30). It introduces a regularized model framework to control overfitting and improve performance. Regularization includes a cost term that encourages many variable weights to approach zero, thereby reducing overfitting. Penalty regularization effectively mitigates overfitting without sacrificing predictive power (31). An essential aspect of boosting is that each new model is built upon the errors of the previous iterations, with each decision tree adjusted based on the model's residuals. By leveraging these ensemble learning techniques, we aim to leverage the collective strength of multiple weak models to create a more accurate and robust prediction model for LOS prediction.

XGBoost also includes several essential hyperparameters, and their descriptions can be found in Table 4. The XGBoost objective function incorporates a regularization concept, which plays a crucial role in selecting predictive functions and managing the model's complexity. By combining the loss function with the regularization term, we obtain the complete objective function of XGBoost. This objective function effectively balances the model's predictive power, governed by the loss function, and its simplicity, controlled by the regularization term. We can formally express the objective function of XGBoost as demonstrated in Equation (1):

(1) $$Obj=\overset{n}{\underset{i=1}{\sum }}\;L({\hat{y}}_{i},\;y_{i})+\overset{k}{\underset{i=1}{\sum }}\;R(f_{i}\;)\;$$

**Table 4:** XGBoost classifier parameters.

Parameter	Default	Description
learning_rate	0.3	Shrink the weights on each step
n_estimators	100	Number of trees to fit.
objective	binary: logistic	logistic regression for binary classification
booster	gbtree	Select the model for each iteration
nthread	max	Input the system core number
min_child_weight	1	Minimum sum of weights
max_depth	6	Maximum depth of a tree.
gamma	0	The minimum loss reduction needed for splitting
subsample	1	Control the sample's proportion
colsample_bytree	1	Column's fraction of random samples
reg_lambda	1	L2 regularization term on weights
reg_alpha	0	L1 regularization term on weights

where *L* represents the loss function, which assesses the model's compatibility with the training data. The predicted label is denoted by f, while y represents the actual label. Furthermore, *R(f)* plays a pivotal role in penalizing the complexity of the functions within the training tree. In this algorithm, we also use the scikit-learn package of python for analysis.

#### 2.2.3 Model's performance analysis

Commonly used regression performance metrics have been employed to evaluate the prediction results of the model. The metrics utilized for assessment include mean absolute error (MAE), root mean square error (RMSE), and mean absolute percentage error (MAPE) (32). In the context of these metrics, lower values of MAE and RMSE indicate better performance with fewer errors. These metrics are defined as in Equations 2–4:

(2) $$MAE=\;\frac{1}{N}\overset{N}{\underset{I=1}{\sum }}\;|{\hat{y}}_{i}-y_{i}|$$

(3) $$RMSE=\sqrt{\;\frac{1}{N}\overset{N}{\underset{I=1}{\sum }}\;{\left({\hat{y}}_{i}-y_{i}\right)}^{2}}$$

(4) $$MAPE=\;\frac{1}{N}\overset{N}{\underset{I=1}{\sum }}\;(\frac{\left({\hat{y}}_{i}-y_{i}\right)}{y_{i}},\;100)$$

In the equations provided, *y_i_* presents the observed LOS and ${\hat{y}}_{i}$ represents the the predicted LOS for a given sample. *N* stands for the total number of samples in the dataset. It's important to note that all these metrics are oriented negatively, meaning that a lower value indicates a better model performance.

The MAE and RMSE are used to measure the model's average prediction error of remaining useful life. The values of these two metrics can range from 0 to positive infinity. RMSE is computed as the square root of the mean squared difference between the true LOS and the predicted LOS. It is always nonnegative, and an RMSE of zero suggests a perfect fit to the data. However, achieving an RMSE of zero is rarely attainable in practice and can often indicate overfitting of the model to the training data.

On the other hand, the MAPE is a variant of MAE. It quantifies the absolute error normalized over the data and is a widely used metric due to its interpretability. Specifically, a MAPE of 30% means that the model, on average, approximates the target value with an accuracy of 70%, calculated by subtracting 30% from 100%. This provides a straightforward way to understand the model's performance in terms of percentage accuracy.

#### 2.2.4 Variable importance

Traditional methods such as stepwise regression, grey relational analysis, or correlation analysis can be used to evaluate the explanatory power of independent variables on the dependent variable. However, when multiple independent variables exhibit multicollinearity, these methods may not perform well, resulting in the retention of certain variables that negatively affect cost prediction, thereby increasing estimation errors. Using variable importance methods can more accurately select independent variables for highly correlated data and small sample sizes. He et al. (33) proposed that not only reflects the importance of independent variables to the model but also the extent to which the dependent variable is explained. The value of the *j-th* independent variable for the dependent variable can be expressed *V* as in Equation (5):

(5) $$V=\sqrt{\frac{k}{Rd\;(Y;\;t_{1}\ldots t_{h})}\sum_{i=1}^{h}Rd\;(Y;\;t_{1})w_{ij}^{2}}$$

Here, *k* represents the number of independent variables, *w_ij_* denotes the weight of the *i*-th variable in the *j*-th component, *h* indicates the total number of components, *Y* represents the dependent variable, *Rd (Y;t_1_,…t_h_)* denotes the explanatory power of *t_1_* to *t_h_* on *Y*, and *Rd (Y;t_1_)* represents the explanatory power of *t_i_* on *Y* as shown in Equations (6–7).

(6) $$Rd\;(Y;\;t_{i})=r^{2}(Y;\;t_{i})$$

(7) $$Rd\;(Y;\;t_{1}\ldots t_{h})=\sum_{t=1}^{h}Rd\;(Y;\;t_{i})$$

The correlation coefficient between the principal component *t_i_* and the dependent variable Y is denoted as $r(Y;t_{1})$.

Since the explanatory power of *x_j_* on depends on its transformation through *t_j_*, the stronger the explanatory power of *t_i_* on *Y*, the stronger the explanatory power of *x_j_* on *Y* will be, and the larger the value will become. The value represents the extent to which an independent variable affects the model's fit (33). Therefore, if all independent variables have the same explanatory power for *Y*, the value should be 1; otherwise, a lower value indicates weaker explanatory power of that independent variable on the dependent variable. A value less than 1 suggests that the variable is less critical and may be considered for removal. However, it is not recommended to eliminate all independent variables with values less than 1 automatically; instead, it is advisable to discard the variable with the smallest value first. If the predictive model has been optimized, this process can be repeated on more minor variables until no further optimization occurs. Therefore, the method can be used to select independent variables.

## 3 Results

### 3.1 Prediction results analysis

The model that achieved the lowest values in these metrics was considered the best prediction model. Table 5 presents Wuhan city household registration results, utilizing three linear regression models and three integrated learning models. The integrated learning models outperformed the linear regression models in terms of prediction accuracy. Integrated learning incorporates individual classifiers, which provide reasonable bounds and reduce the overall error rate, resulting in improved prediction results. Notably, XGB performed the best among the six algorithms, consistently achieving lower errors. This model is capable of parallel computation, further minimizing errors.

**Table 5:** The results from different models for prediction LOS of household registration in Wuhan before and during the COVID-19.

Methods	Years	Medical insurance			Without medical insurance		
		MAE	RMSE	MAPE	MAE	RMSE	MAPE
LR	2017	37.15	6.09	0.48	45.96	6.78	0.57
	2018	34.06	5.84	0.6	37.83	6.15	0.64
	2019	28.28	5.32	0.62	37.63	6.13	0.68
	2020	56.21	7.5	0.71	64.05	8	0.75
	2021	23.01	4.8	0.74	29.09	5.39	0.81
	2022	40.65	6.38	0.84	51.9	7.2	0.91
LA	2017	38.04	6.17	0.48	46.29	6.8	0.57
	2018	34.56	5.88	0.61	38.02	6.17	0.65
	2019	28.79	5.37	0.65	37.92	6.16	0.7
	2020	56.45	7.51	0.72	64.42	8.03	0.76
	2021	23.55	4.85	0.77	29.51	5.43	0.82
	2022	40.82	6.39	0.84	51.83	7.2	0.9
RR	2017	37.16	6.1	0.48	45.96	6.78	0.57
	2018	34.05	5.84	0.6	37.82	6.15	0.64
	2019	28.28	5.32	0.62	37.63	6.13	0.68
	2020	56.2	7.5	0.71	64.05	8	0.75
	2021	23.01	4.8	0.74	29.09	5.39	0.81
	2022	40.62	6.37	0.84	51.81	7.2	0.9
AB	2017	31.76	5.64	0.41	37.89	6.16	0.5
	2018	25.16	5.02	0.44	32.16	5.67	0.58
	2019	21	4.58	0.46	32.65	5.71	0.6
	2020	40.28	6.35	0.5	51.53	7.18	0.63
	2021	15.23	3.9	0.47	19.69	4.44	0.61
	2022	19.71	4.44	0.51	24.57	4.96	0.63
BG	2017	32.25	5.68	0.4	38.5	6.21	0.48
	2018	24.92	4.99	0.42	32.28	5.68	0.55
	2019	23.02	4.8	0.43	30.92	5.56	0.55
	2020	40.23	6.34	0.47	52.49	7.25	0.57
	2021	15.32	3.91	0.45	19.91	4.46	0.56
	2022	23.73	4.87	0.52	30.31	5.51	0.62
XGB	2017	28.62	5.35	0.34	37.35	6.11	0.4
	2018	23.67	4.87	0.36	30.13	5.49	0.45
	2019	20.34	4.51	0.37	32.54	5.7	0.45
	2020	35.65	5.97	0.41	45.64	6.76	0.49
	2021	13.4	3.66	0.38	17.34	4.16	0.46
	2022	17.37	4.17	0.43	21.81	4.67	0.51

**Figure 2:**
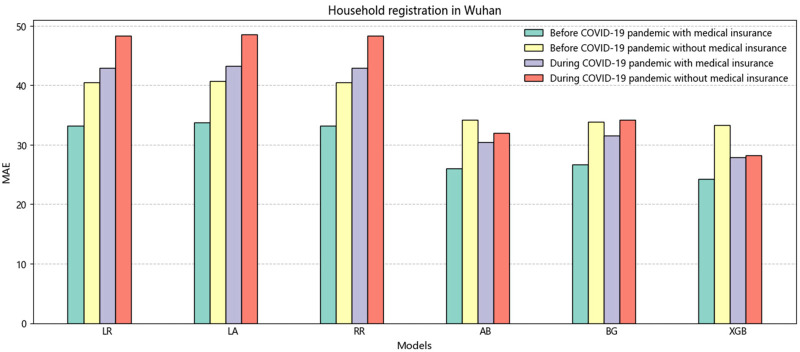
The histogram of the average value of MAE is non-household registration in Wuhan before and during the COVID-19.

Furthermore, when comparing the periods before and during the COVID-19 pandemic, we observed a noticeable increase in errors associated with medical insurance. On average, there was a threefold increase in errors, which can be attributed to many people staying at home during the pandemic and not utilizing their medical insurance. Comparing individuals who use medical insurance with those who do not, we found that the error rate was lower for those who used medical insurance. This trend is particularly evident in Figure 2, where the MAE indicator supports this finding. Moreover, when considering RMSE, it is obvious that the errors were lower for individuals with medical insurance than those without. Specifically, the errors were 0.79 for 2020, 0.5 for 2021, and 0.5 for 2022, respectively. These results suggest that medical insurance is a critical variable in predicting LOS.

**Figure 3:**
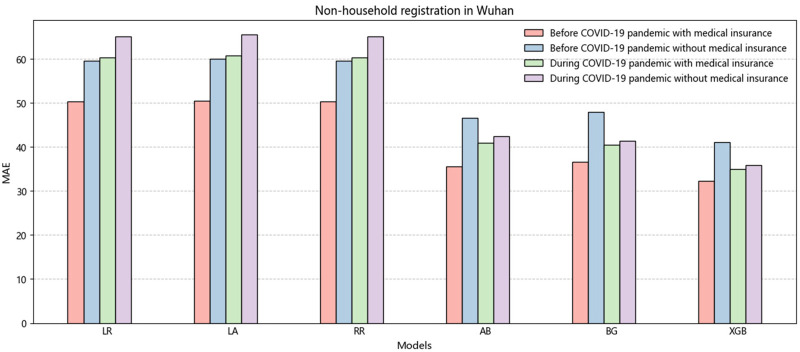
The histogram of the average value of MAE is non-household registration in Wuhan before and during the COVID-19.

Table 6 presents the results for individuals with non-Wuhan household registration, and the overall findings are consistent with those shown in Table 5. The results indicate that the XGB model, which represents integrated learning, outperforms the linear regression model regarding prediction accuracy. The minor error obtained by XGB further emphasizes the superiority of this model, with metrics such as MAE (26.33), RMSE (5.13), and MAPE (0.44). Figure 3 shows a higher overall error for non-Wuhan domiciles than for Wuhan domiciles. This disparity arises from the fact that China's medical insurance reimbursement system is based on domicile. Non-Wuhan domiciles incur higher medical expenses when seeking treatment at Wuhan Central South Hospital, increasing patient costs. It is worth noting that many countries, including China, have implemented adequate measures to expand medical coverage. However, due to regional economic disparities, the percentage of medical insurance reimbursement varies across different areas (34). We recommend that China focuses on improving its medical insurance system in the future.

**Table 6:** The results from different models for prediction LOS of non-household registration in Wuhan before and during the COVID-19.

Methods	Years	Medical insurance			Without medical insurance		
		MAE	RMSE	MAPE	MAE	RMSE	MAPE
LR	2017	48.76	6.98	0.53	58.11	7.62	0.59
	2018	46.23	6.8	0.6	57.7	7.6	0.64
	2019	55.72	7.46	0.7	62.9	7.93	0.75
	2020	70.53	8.4	0.78	83	9.11	0.84
	2021	54.05	7.35	0.87	60.71	7.79	0.94
	2022	44.07	6.64	0.9	51.58	7.18	0.95
LA	2017	48.19	6.94	0.53	58.4	7.64	0.59
	2018	47.03	6.86	0.61	57.82	7.6	0.65
	2019	56.16	7.49	0.71	63.51	7.97	0.76
	2020	70.88	8.42	0.79	83.26	9.12	0.84
	2021	54.58	7.39	0.87	61.29	7.83	0.94
	2022	44.92	6.7	0.91	52.07	7.22	0.95
RR	2017	48.68	6.98	0.53	58.06	7.62	0.59
	2018	46.25	6.8	0.6	57.69	7.6	0.64
	2019	55.72	7.46	0.7	62.9	7.93	0.75
	2020	70.52	8.4	0.78	83	9.11	0.84
	2021	54.04	7.35	0.87	60.71	7.79	0.94
	2022	44.07	6.64	0.9	51.58	7.18	0.95
AB	2017	38.95	6.24	0.41	47.31	6.88	0.5
	2018	37.51	6.12	0.45	49.53	7.04	0.55
	2019	30	5.48	0.48	42.89	6.55	0.6
	2020	43.92	6.63	0.53	59.01	7.68	0.69
	2021	33.05	5.75	0.55	37.08	6.09	0.68
	2022	24.57	4.96	0.57	31.18	5.58	0.71
BG	2017	41.78	6.46	0.4	52.26	7.23	0.48
	2018	38.1	6.17	0.43	49.87	7.06	0.53
	2019	30.03	5.48	0.46	41.78	6.46	0.57
	2020	41.58	6.45	0.5	55.49	7.45	0.63
	2021	30.26	5.5	0.52	37.12	6.09	0.62
	2022	24.97	5	0.53	31.23	5.59	0.65
XGB	2017	36.96	6.08	0.36	44.37	6.66	0.41
	2018	34.02	5.83	0.38	43.01	6.56	0.46
	2019	25.96	5.1	0.41	35.93	5.99	0.49
	2020	38.15	6.18	0.44	49.93	7.07	0.54
	2021	26.33	5.13	0.44	31.57	5.62	0.53
	2022	19.68	4.44	0.45	26.08	5.11	0.55

Additionally, our research reveals that patients' insurance spending on medical visits has increased significantly because of the significant impact of the COVID-19 pandemic on Wuhan. This situation has led patients to pay greater attention to their health, resulting in increased medical insurance expenditures and longer LOS. Conversely, it is evident that after 2020, the situation in Wuhan gradually improved as the pandemic subsided, and the country's economy also recovered. Consequently, more investment was made in medical insurance, benefiting more patients.

### 3.2 Variable importance analysis

**Figure 4:**
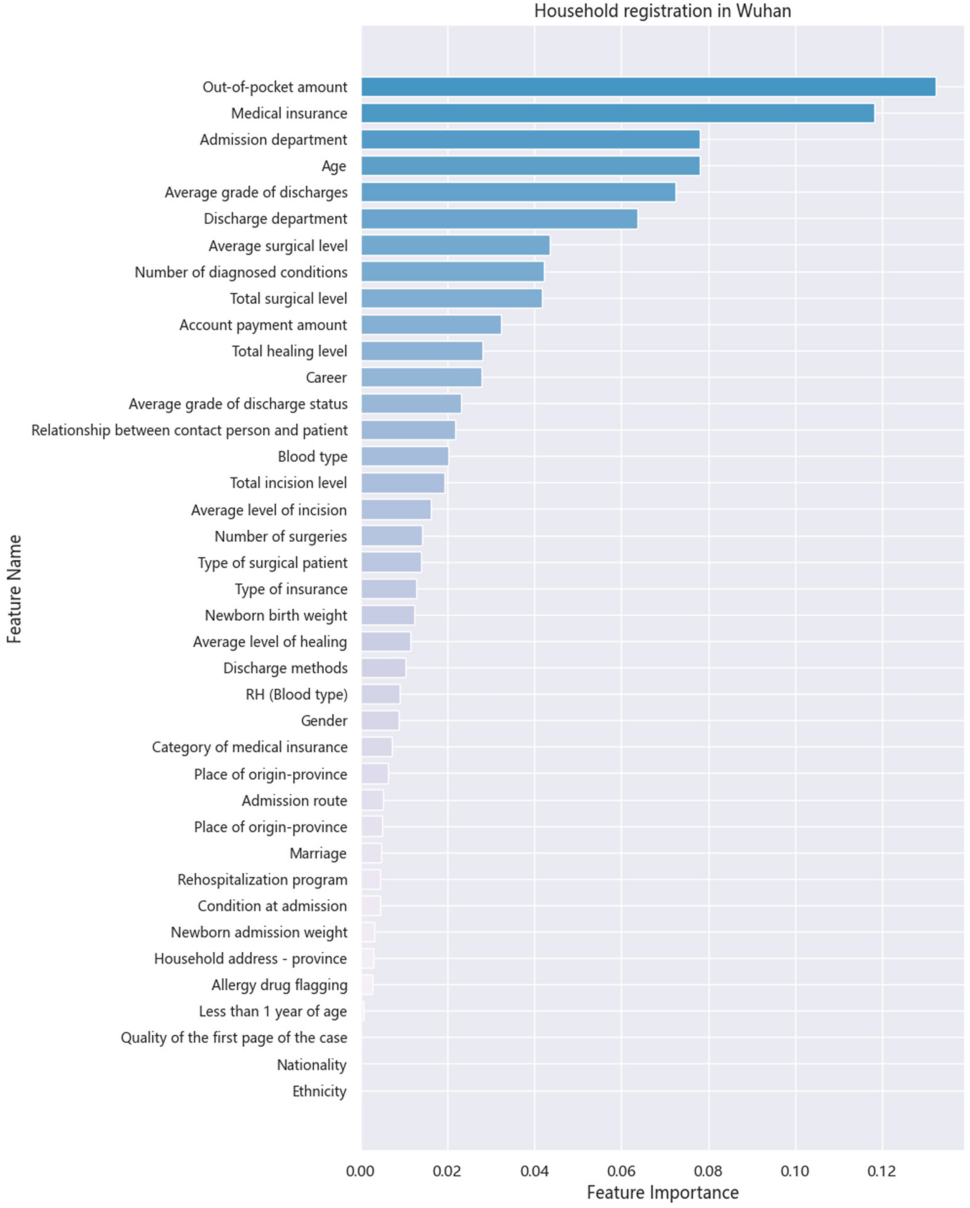
Importance of each variable for household registration.

**Figure 5:**
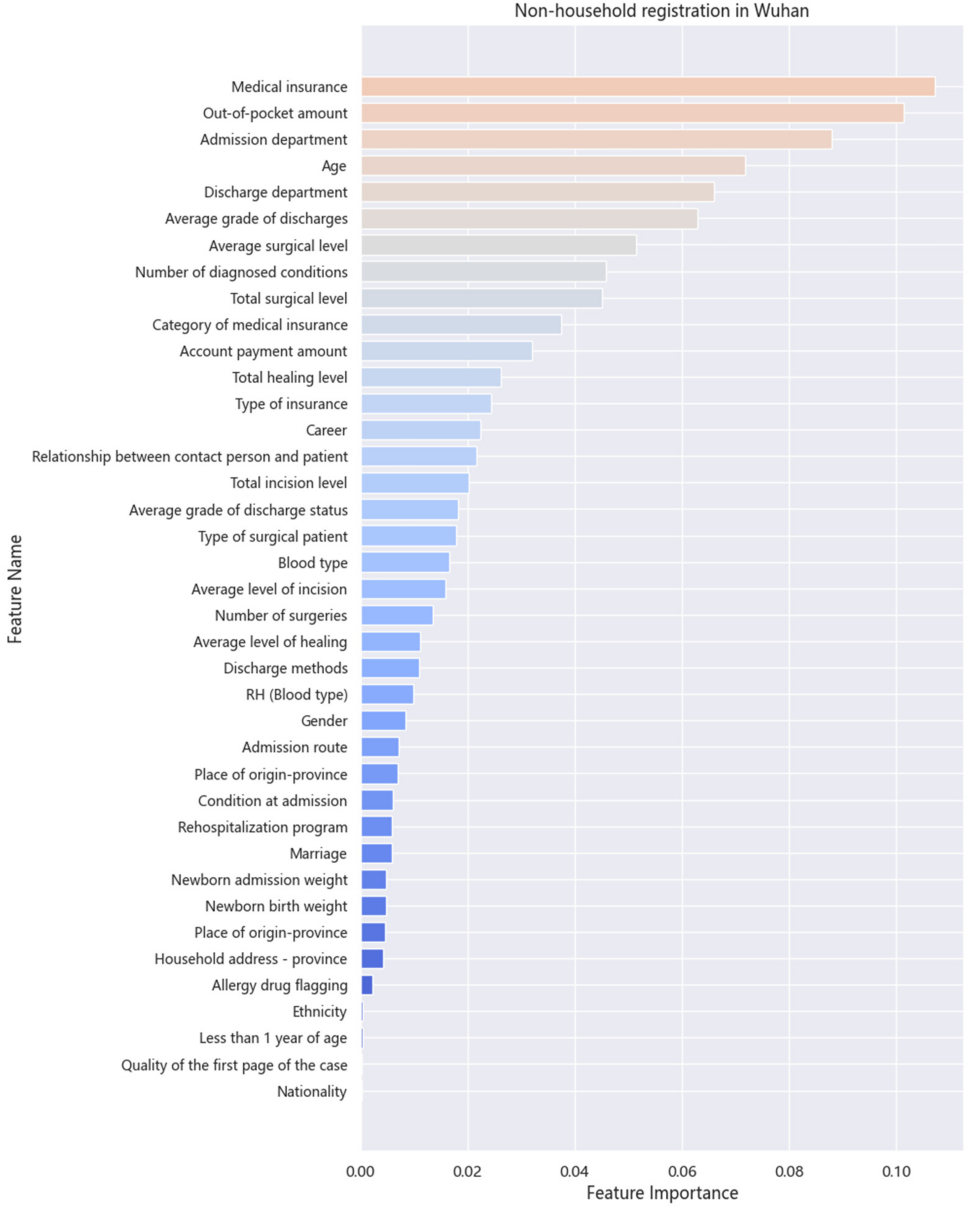
Importance of each variable for non-household registration.

Figures 4, 5 display the contribution of each variable to the model's gain in patients with Wuhan and non-Wuhan household registrations. The highest percentage indicates the importance of the prediction. The most influential features in the XGB model for LOS prediction are medical insurance and the out-of-pocket amount, in that order. As Chen et al. (30) mentioned in the boosting tree model, the gain of each feature is considered, and the average increase provides the overall vision of the entire model.

In China, patients still prioritize tertiary hospitals because they believe large comprehensive hospitals offer better medical resources. This phenomenon makes managing the availability of hospital beds a challenging task, especially when the actual hospital service level needs to meet expectations (35). Zhongnan Hospital of Wuhan University is one of the largest tertiary hospitals in Wuhan. Many patients have multiple comorbidities, and the average age is relatively high. These factors make it difficult to predict the LOS and partially compensate for the limitations of single-center studies. Furthermore, the imbalance in the Chinese economy leads to different reimbursement rates for medical insurance. This article measures the differences in medical insurance between Wuhan and non-Wuhan household registrations. It finds that patients without Wuhan household registration spend more money. This paper highlights the need for the government to effectively allocate medical insurance resources and provide adequate care for patients in more remote areas.

The model identifies medical insurance as the most influential variable, indicating that the type and extent of coverage significantly impact LOS. Patients with comprehensive medical insurance may experience shorter hospital stays due to reduced financial barriers and quicker access to necessary treatments. This finding underscores the importance of economic coverage in healthcare. By prioritizing insurance as a predictor, the model suggests that patients' experiences and outcomes are closely tied to their insurance status, prompting considerations for policymakers to enhance insurance equity, especially for economically disadvantaged groups.

The second most important feature is out-of-pocket expenses, highlighting how financial strain influences patient behavior and treatment outcomes. Patients facing higher personal costs may delay care or seek fewer intensive treatments, potentially extending their LOS. The model's emphasis on this variable reveals a critical connection between financial responsibility and healthcare decisions. This suggests that interventions to reduce out-of-pocket costs, such as subsidies or better reimbursement policies, could positively influence patient outcomes by shortening LOS. The differentiation between Wuhan and non-Wuhan household registrations points to the geographical and economic disparities in healthcare access and spending. Patients without Wuhan household registration incur higher costs, reflecting the inequalities in medical resource distribution and insurance coverage across regions. This insight provides a clear understanding of how socioeconomic factors shape healthcare experiences. It indicates that patients from non-Wuhan areas face additional barriers to care, emphasizing the need for targeted policies to ensure equitable access to healthcare resources.

The discussion of patient preference for tertiary hospitals highlights a broader cultural and systemic phenomenon in China, where patients perceive larger hospitals as offering better care. This preference can lead to higher resource demand in these institutions, complicating bed management and potentially leading to increased LOS. The model's results can inform hospital management strategies. Understanding that patients prioritize comprehensive hospitals can guide administrators in resource allocation and operational plans to meet demand without compromising care quality. The analysis thoroughly explains how these factors affect patient outcomes by highlighting critical variables such as medical insurance, out-of-pocket expenses, and geographic disparities. This interpretative clarity is essential for guiding policymakers and healthcare administrators in making informed decisions to enhance healthcare access, reduce LOS, and improve overall patient care quality.

## 4 Discussion

### 4.1 Comparative analysis

The main findings of this study emphasize the crucial role that machine learning plays in predicting and managing patients' LOS, especially after incorporating an analysis of variable importance (36). By using these machine learning models, hospitals can better predict patient LOS, thereby optimizing resource allocation, improving healthcare service efficiency, and reducing medical costs. However, the study also reveals that during this process, the number of admissions decreased (37). This finding aligns with other studies in the literature, which indicate a significant increase in hospital admissions in Wuhan during the COVID-19 pandemic (38). Notably, the accuracy of predictions made by integrated learning methods significantly surpasses that of traditional machine learning methods. Integrated learning enhances the stability and precision of predictions by combining the strengths of multiple models (39). This result not only demonstrates the potential of integrated learning in handling complex medical data but also lays a foundation for its broader application in the healthcare field in the future.

Furthermore, the predictive results highlight that patients with Wuhan residency exhibited more significant medical needs and complexities during the pandemic compared to non-residents (40). This indicates that Wuhan's healthcare system faced more significant challenges during the pandemic, requiring more complex and intensive medical interventions for residents. Consequently, hospital admission decisions were more cautious and precise, consistent with findings from other studies (41). From both economic and clinical perspectives, the COVID-19 pandemic has driven the development of new management strategies, particularly in the management of stroke patients, providing valuable insights for addressing similar public health crises in the future. Compared to our previous analysis, which focused solely on pre-pandemic hospitalizations, the relative increase in weight during the pandemic has been further confirmed (42). This suggests that during a pandemic, the burden on the healthcare system and the allocation of resources become more critical than ever before. For patients, the results of linear regression analysis indicate a significant reduction in LOS, leading to increased bed utilization. This finding suggests that the healthcare system's response capabilities were significantly enhanced during the pandemic.

Eftekhar et al. (43) Studies in the U.S. have extensively applied machine learning models, such as logistic regression and neural networks, to predict LOS in diverse healthcare settings. These studies often emphasize the role of clinical variables, such as comorbidities, and demographic factors, while highlighting challenges in data standardization across hospitals. Nowroz et al. (44) focus on integrating electronic health records with ML techniques to refine LOS predictions. European studies often benefit from robust healthcare data systems, enabling comparative analyses across institutions, but face challenges related to GDPR compliance in data sharing. Al-Hanawi (45) usually addresses the unique characteristics of healthcare systems, such as the influence of seasonal pilgrimages on hospital admissions and LOS predictions. Compared with other countries, our research still highlights the background of local research in China, emphasizing the unique socioeconomic, cultural, and policy-driven factors that influence the study context. For instance, the distinct healthcare policies, population density, and the government's robust public health interventions during the COVID-19 pandemic create a research environment that is notably different from that of Western or other Asian countries. Furthermore, integrating traditional Chinese medicine with modern healthcare practices, the rapid digitalization of healthcare services, and China's unique economic recovery strategies provide a rich and distinct backdrop for our analysis. These factors make our findings particularly relevant to China's context and offer insights that could inspire comparative studies in other regions.

### 4.2 Implication for research

In this study, we harnessed machine learning techniques to predict patients' hospitalization durations before and during the pandemic for household registration in Wuhan and non- household registration in Wuhan. Beyond accurately forecasting patients' LOS, this paper contributes to the body of literature on sustainable healthcare and clinical decision support systems across two critical dimensions: theory and methodology. Theoretically, we introduced an innovative paradigm for predicting hospital LOS that involves validating patients' LOS both before and during the pandemic. In terms of methodology, we strongly emphasized the significance of the XGB prediction method.

We underscored the importance of our approach by conducting comparative analyses across different timeframes, shedding light on the practicality and realism of constructing predictive models. Additionally, our approach to variable selection underscores the significance of treating healthcare variables as interrelated records. Notably, demographic information about patients emerged as one of the most pivotal predictors of LOS (46). The role of patients' health insurance status is particularly important as a critical predictor, emphasizing the need for greater collaboration and information sharing between governmental bodies and healthcare institutions. Such partnership can yield more comprehensive medical histories, facilitating easier tracking and refinement of predictions. Our research accentuates the pressing need for hospitals to enhance their predictive capabilities. By doing so, they can access more data to inform decisions across various facets of patient healthcare provision. As our discussions have shown, these efforts have the potential to bring economic advantages to hospitals and improvements in health outcomes, enhanced patient service, and, ultimately, more sustainable operations.

### 4.3 Implication for practice

Machine learning enables us to leverage patient information and hospitalization data to effectively determine clinical strategies at an early stage (47), thereby optimizing LOS. Using machine learning algorithms, relevant healthcare administrators, medical professionals, patients, and their families can benefit from guidance and support (48). This approach facilitates the provision of routine care and efficient information, including social medical insurance funding and treatment options. One significant advantage of accurately predicting LOS during a pandemic is that it allows hospital administrators to plan the required beds and staff more effectively. Furthermore, it enables identifying patients who may require a longer LOS, thus directly improving the quality of care.

Additionally, it aids in the rational allocation of scarce resources. To evaluate the performance of our prediction model, we employ metrics such as MAE, MAPE, and RMSE, among others. These metrics provide a more direct and convincing assessment for physicians compared to other methods. Our proposed model can assist clinicians in addressing various challenges associated with timely patient discharge following disease outbreaks. While machine learning has demonstrated remarkable performance in handling image, text, and speech data (49), it may encounter overfitting issues when dealing with unstructured data. XGB has emerged as the most accurate and effective algorithm among the models we have explored.

Machine learning models are prone to biases based on the data they are trained on. If the training data predominantly represents a specific population (e.g., patients from Wuhan), the model may generalize poorly to other populations, leading to inequitable treatment recommendations. This raises ethical concerns about fairness and equity in healthcare, as marginalized or underrepresented groups may not receive optimal care based on model predictions, potentially exacerbating existing disparities. If healthcare providers rely solely on machine learning outputs without considering clinical judgment, it may lead to suboptimal care or neglect of individual patient needs. Current policies may need to be better equipped to handle the complexities and implications of algorithmic decision-making in clinical settings. The study highlights disparities in medical insurance and access to healthcare resources, suggesting that financial inequities influence LOS.

### 4.4 Limitation and future work

The input features of our model heavily rely on admission predictions, which can be challenging to generate, especially when hospitals have patients with diverse diseases. However, leveraging past information on LOS can help reduce uncertainty. For instance, patients with different diseases often exhibit distinct peak occupancy times before and during the COVID-19 pandemic. Therefore, our model utilizes accurate bed admission predictions as a crucial step in LOS prediction. Another key aspect is understanding the causes of regional and sub-regional heterogeneity in patients. We can enhance our understanding of predictive models by matching this information with known variations in factors such as socioeconomic status and comorbidities. Future research could focus on further exploring the factors that influence patient prognosis.

In our study, we utilized six machine learning algorithms, and the results demonstrated that XGB exhibited solid predictive performance, especially in specific years. Moreover, the critical variables computed by the algorithms before and during the COVID-19 pandemic improved the alignment of the model-predicted bed occupancy with the timeline (50). However, this assumption may lead to overestimating bed occupancy in other cases. With the advancement of artificial intelligence techniques, we can incorporate deep learning prediction methods in future iterations.

Regarding the data, we acknowledge that we limited ourselves to collecting data from a single hospital during the COVID-19 pandemic. As time has progressed, there may have been changes in factors such as discharge policies, clinical care practices, and admission statuses, which could impact the accuracy of our predictions. Additionally, the small sample size and inherent randomness of the results pose challenges in estimating these temporal changes accurately. However, considering our focus on LOS for COVID-19 patients, we did not exclude them from our data. To ensure robustness, future efforts should expand the data sample to include more hospital data.

We know that the data we are using now needs to be improved, which may constrain the generalizability and scope of our findings. However, as our research progresses, we are committed to incorporating a larger and more diverse dataset to enhance the robustness and accuracy of our analysis. By expanding the volume and variety of data, we aim to explore additional dimensions of the problem, uncover deeper insights, and validate our conclusions across broader scenarios. This approach will not only strengthen the reliability of our results but also pave the way for a more comprehensive understanding of the subject matter.

## 5 Conclusion

Machine learning is a valuable tool that can aid hospital administrators in making early clinical decisions, mitigating associated risks, and optimizing the allocation of healthcare resources. This study focuses on predicting LOS in hospitals before and during the COVID-19 pandemic. By accurately assessing the patient load, hospitals can improve the management of patient admissions, leading to reduced fluctuations in bed occupancy. In this study, we examined six different algorithmic survival models for predicting LOS. These models allow for a deeper understanding of patient-specific LOS distributions. Our findings indicate that the patient's medical insurance status is a critical predictor variable, regardless of whether the patients are from Wuhan or non-Wuhan regions. Moreover, the selected models demonstrate variations in prediction performance. However, we have also observed that incorporating features derived from text and images in electronic medical records can enhance LOS prediction. Exploring this avenue further could be a potential direction for future research.

## Data Availability

The original contributions presented in the study are included in the article/Supplementary Material, further inquiries can be directed to the corresponding author.
